# Association Between Ov16 Seropositivity and Neurocognitive Performance Among Children in Rural Cameroon: a Pilot Study

**DOI:** 10.1007/s40817-021-00111-z

**Published:** 2021-09-07

**Authors:** Joseph Nelson Siewe Fodjo, Wepnyu Y. Njamnshi, Leonard Ngarka, Leonard N. Nfor, Constance Ayuk, Noelar N. Mundih, Hilda T. Ekwoge, Kevin Nganchfu, Kongnyu G. Njamnshi, Rachel Yerema, Pernelle Ngoundjou, Edward Awasume, George Ashu, Earnest N. Tabah, Robert Colebunders, Alfred K. Njamnshi

**Affiliations:** 1 Global Health Institute, University of Antwerp, Antwerp, Belgium; 2 Brain Research Africa Initiative (BRAIN), Yaoundé, Cameroon; 3 Neuroscience Lab, Faculty of Medicine & Biomedical Sciences, The University of Yaoundé I, Yaoundé, Cameroon; 4 Department of Neurology, Yaoundé Central Hospital, Yaoundé, Cameroon; 5 Faculty of Medicine & Pharmaceutical Sciences, The University of Dschang, Dschang, Cameroon; 6 Buea Regional Hospital, Buea, Cameroon; 7 HILPharma Organization, Yaoundé, Cameroon; 8 Ministry of Public Health, Yaoundé, Cameroon

**Keywords:** Ov16, Onchocerciasis-associated epilepsy, Neurocognitive assessment, Cameroon

## Abstract

Infection with *Onchocerca volvulus* was recently reported to increase the risk for epilepsy in Cameroonian children. We investigated whether infection with *O. volvulus* may alter the cognitive function of children who may or may not develop epilepsy later in their lifetime. Using rapid diagnostic tests, we determined the presence of Ov16 antibodies in 209 school-aged children without epilepsy recruited from three Cameroonian villages, as a proxy for onchocerciasis exposure. In addition, the neurocognitive performance of these children was assessed using a battery of validated tools. Participants were aged 6–16 years, and 46.4% were Ov16 seropositive. Upon standardizing age-specific neurocognitive scores and investigating predictors of neurocognitive performance using multiple linear regression models (adjusted for gender, education level, previous ivermectin use, and anthropometric parameters), we found that being Ov16-positive was significantly associated with reduced semantic verbal fluency (estimate –0.38; 95% confidence interval –0.65 to –0.11; *p* = 0.006) and lower scores on the International HIV Dementia Scale (estimate –0.31; confidence interval –0.56 to –0.04; *p* = 0.025). Furthermore, an increasing frequency of past ivermectin use was associated with increased neurocognitive scores. Our findings suggest that exposure to *O. volvulus* may affect neurocognitive performance of children.

**Supplementary Information:**

The online version contains supplementary material available at 10.1007/s40817-021-00111-z.

## Introduction

Onchocerciasis (river blindness) is a tropical filariasis caused by *Onchocerca volvulus* and transmitted to humans via the blackfly vector (Simulidae) (Burnham G 1998). There is accumulating epidemiological evidence suggesting an association between onchocerciasis and epilepsy, with a 10% increase in onchocerciasis prevalence estimated to produce a 0.4% increase in epilepsy prevalence (Pion et al., 2009). Two cohort studies in Cameroon further showed the temporality of this association, revealing an increased risk to develop epilepsy following childhood infection with *O. volvulus*, in a microfilarial load-dependent manner (Chesnais et al., 2018, 2020). The terms ‘onchocerciasis-associated epilepsy (OAE)’ and ‘river epilepsy’ have been proposed to regroup the onchocerciasis-related seizure disorders frequently encountered in endemic areas (Colebunders et al., 2018a, b). Observations from community-based surveys in onchocerciasis-endemic regions indicate that OAE consists of a wide clinical spectrum involving seizure disorders (nodding syndrome (NS) and other forms of epilepsy), retarded growth (stunting), and delayed development of secondary sexual characteristics (Nakalanga features) (Colebunders et al., 2018a, b; Föger et al., 2017; Raper and Ladkin, 1950; Siewe Fodjo 2019a, b, c).

OAE constitutes an important burden for families and communities, particularly because children and adolescents are more prone to developing the disease. Indeed, OAE usually starts between the ages of 3 and 18 years (Colebunders et al., 2018a, b). Nevertheless, early detection and proper management of OAE can significantly improve the quality of life of the patients and prevent complications (Idro et al., 2014; Siewe Fodjo et al., 2019a). A follow-up study of persons with NS in Uganda suggests that this condition may include prodromal features (excessive sleepiness, slowing down of activities, decline in comprehension, blank staring) which gradually progress towards more conspicuous, convulsive manifestations; the reported duration between the onset of these prodromal symptoms and development of seizures varied from a few weeks to 2 years (Idro et al., 2018). Indeed, previous studies revealed that persons with OAE often show signs of cognitive impairment (Colebunders et al., 2018a, b; Siewe Fodjo et al., 2019a, b, c). Furthermore, in a recent case–control study in onchocerciasis-endemic communities in Cameroon in Cameroon, we found that cognitive impairment (assessed using a comprehensive neuropsychological test battery) was significantly more prevalent in persons with epilepsy (PWE) than in age- and sex-matched controls (Njamnshi et al., 2020). However, it is still unclear whether the cognitive symptoms observed in persons with OAE preceded seizure onset, or were solely consequences of the repeated epileptic episodes. We therefore decided to assess the cognitive function of children without epilepsy in onchocerciasis-endemic villages of Cameroon and to investigate possible associations with previous exposure to *O. volvulus.*

### Study Hypothesis

We hypothesized that, besides causing epileptic seizures as demonstrated by previous studies, *O. volvulus* may also induce neurocognitive disorders in exposed children who may or may not develop OAE.

## Methods

### Study Setting

**Fig. 1:**
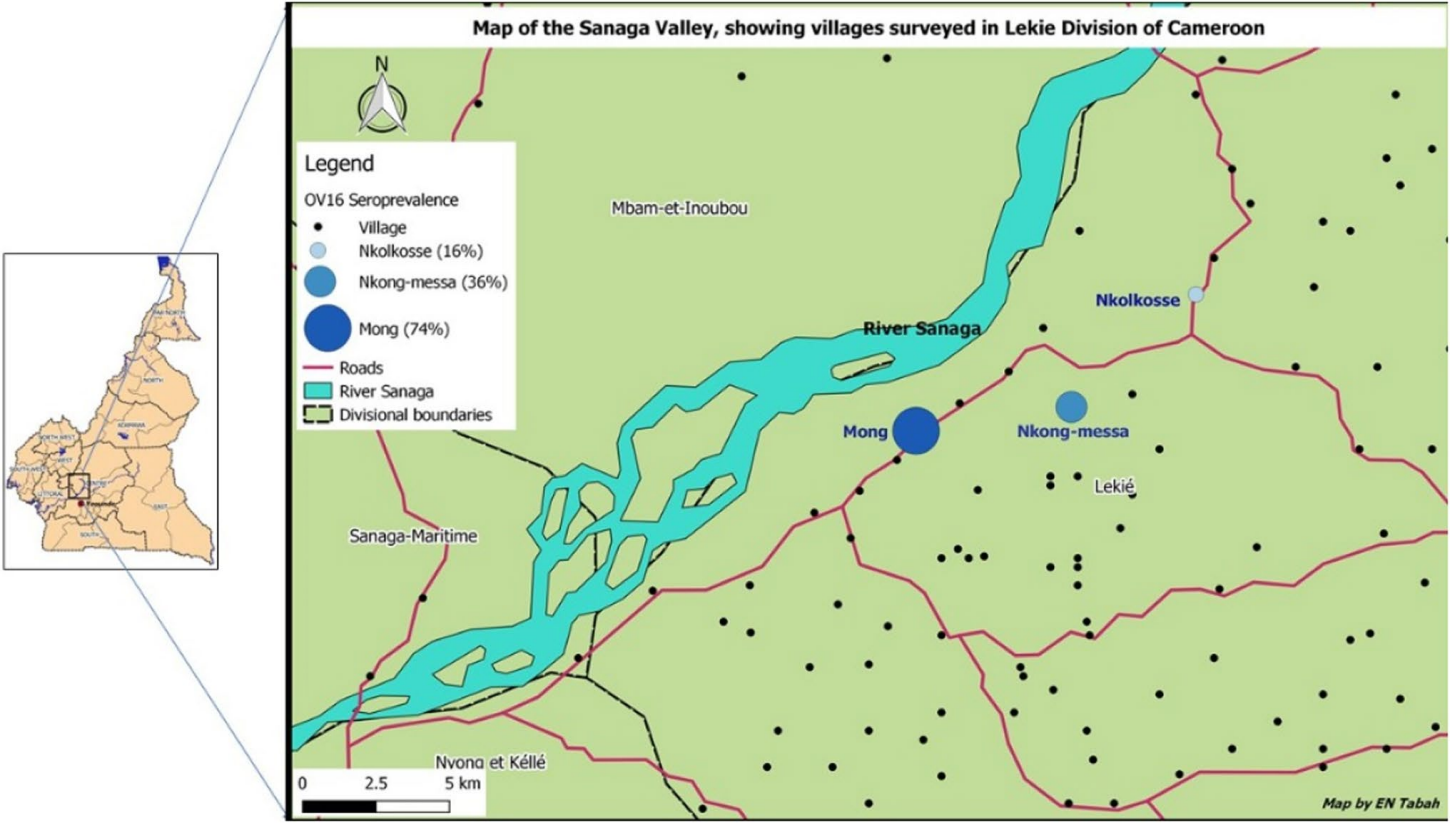
Map showing the location of the three study villages

The study was conducted in three onchocerciasis-endemic villages (Mong, Nkongmessa, and Nkolkosse) in the Lékié division, Centre region, Cameroon Fig. 1. The main blackfly breeding site in this part of the country is the Sanaga River; the distance from the river to the study villages is 2.6 km, 4.5 km, and 6.5 km for Mong, Nkongmessa, and Nkolkosse, respectively. This area is also endemic for loiasis, which has marred the effectiveness of onchocerciasis control using mass treatment with ivermectin (Gardon et al., 1997). The last ivermectin distribution campaign in this area took place in June 2019, almost 2 months before the start of our study. The setting is essentially rural, with farming and petty trading being the main activities. All three study sites were accessible via motorable roads and were close (< 5 km) to the Monatélé town, where there is a district hospital as well as primary and secondary schools.

### Study Design

We conducted a cross-sectional study assessing Ov16 seroprevalence as exposure factor and neurocognitive performance as a primary outcome in 6- to 16-year-old children without any known illness. The approach allowed the constitution of two sub-groups (Ov16-positive participants and Ov16-negative participants) which were analyzed following an unmatched case–control design, comparing the cognitive function of children with and without a history of *O. volvulus* infection.

### Study Participants

Children of both sexes, aged between 6 and 16 years and without epilepsy on clinical evaluation were eligible for enrolment in the study. Determination of the non-epileptic status was done by neurologists or physicians with expertise in epilepsy diagnosis. We excluded children whose past histories suggested an abnormal psychomotor development, a past brain insult (caused by perinatal asphyxia, meningitis), and those who were suffering from any known illness at the time of the survey.

### Study Procedures

The study villages were visited a few weeks before the recruitment of participants. Local authorities (chiefs, health personnel) were informed about the study and their collaboration obtained. They were thus able to sensitize the village residents about the research procedures and encouraged them to participate. Between August 2019 and January 2020, the research team organized field expeditions to each of the study villages, where temporary research units were set up at the residence of the village chiefs. The research team that was involved in the fieldwork in the villages was composed of neurologists (LN, LNN, AKN), physicians (NNM, KN, RY, PN, EA, GA), physicians specialized in epilepsy (JNSF), neuroepidemiology (ENT), and neurocognitive assessment (WYN, CA), a doctor in pharmacy (HTE), and a medical student (KGN) who both participated in Ov16 testing. Children from all over the study village came to the chief’s residence accompanied by a parent/guardian. Assenting children with unremarkable past medical histories, who appeared healthy at the time of the survey, and whose accompanying adult provided an informed consent were enrolled consecutively. Upon enrolment, the socio-demographic information and anthropometric data of the participants were collected.

### Neurocognitive Evaluation

Based on published literature, we selected six cognitive tests which were administered to all the participants. Four of these tests (Purdue Pegboard Test, hand movements, semantic verbal fluency test, digit span) had previously been validated and used by our team to assess Cameroonian school-aged children (Ruffieux et al., 2009, 2013). Two members of the research team (WYN and CA) had been trained to carry out these locally validated tests, but extractions of raw test results and compilation into neurocognitive scores were done by a single investigator with special training in neuropsychological testing (WYN). We also administered a pediatric adaptation of the mini-mental state exam (MMSE) (Moura et al., 2017) to all participants, as well as the International HIV Dementia Scale (IHDS) (Sacktor et al., 2005) for its simplicity, ease of administration, and objectivity across different cultures/educational levels as observed during previous research among Cameroonian adults (Njamnshi et al., 2008). The use of a dementia scale in our study served as a proxy to investigate symptoms of encephalopathy in the participating children. Participants were received one after the other in a prepared, quiet space at the chief’s residence, where the neurocognitive tests were administered. Researchers administering the tests were blinded to the Ov16 status of the subjects. The entire procedure lasted for approximately 30 min per participant. The procedures for each test are explained in details in previous publications (Moura et al., 2017; Njamnshi et al., 2008; Ruffieux et al., 2009, 2013; Sacktor et al., 2005) and summarized in the Supplementary Appendix 1.

### Ov16 Testing

All participants were tested for the presence anti-onchocerca antibodies using the SD Bioline Onchocerciasis Ov16 IgG4 rapid diagnostic test (RDT) (Standard Diagnostics, Gyeonggi-do, South Korea). All procedures were followed as per the manufacturer’s instructions, and RDT results were noted for each participant. Two members of the research team were solely responsible for performing Ov16 tests, and all other researchers were blinded to the Ov16 results until data lock.

### Data Analysis

The collected data were entered into Microsoft Excel 2016 spreadsheets and analyzed in R version 3.6.2. Height-for-age (HfA) as well as BMI-for-age (BfA) *z*-scores were calculated, and any participant that fell below − 2 *z*-score based on the World Health Organization growth curves was considered as stunted (low HfA) or underweight (low BfA) (de Onis, 2007). Cutoff values were defined for the administered tests (5th percentile score in each age group) and used to dichotomize neurocognitive outcomes into normal and below normal. The proportions of children with normal and reduced neurocognitive performance across the study groups (Ov16-positive vs Ov16-negative), stratified by age, were compared using the Yates corrected chi-squared test.

Multiple linear regression models were constructed to investigate factors associated with each neurocognitive outcome. We proceeded by first standardizing the dependent variables (neurocognitive performance) by converting the test scores for each age group into to *z*-scores. For the neurocognitive assessments with several sub-components such as the Pegboard test (dominant, non-dominant, and both hands) and digit span (forward and backward), an average test score was obtained prior to standardization. All models were adjusted for gender, previous ivermectin use, education level, and anthropometric parameters. Age was not included as a covariate because it was already taken into account when creating the *z*-scores by age group. *P*-Values less than 0.05 were considered as statistically significant.

### Ethical Considerations

The study protocol was approved by the University of Antwerp in Belgium (No. B300201731362) and the Cameroon National Ethics Committee for Research in Human Health (No. 2018/12/1123/CE/CNERSH/SP). Administrative authorization was granted by the Ministry of Public Health of Cameroon (D034.19/L/MINSANTE/SG/DROS). The collaboration of local authorities was also obtained for the research project. All participating children gave their assent to participate, and a signed informed consent was obtained from adult parents/guardians. The collected data was treated with absolute confidentiality.

## Results

### Characteristics of Enrolled Participants

A total of 209 children were included in the study (48.8% male). Participants’ ages ranged from 6 to 16 years (median = 10 years; IQR 8–12). Of the 198 children with available data on past ivermectin use, 42 (21.2%) reported to have never taken ivermectin before. Only two (1%) participants were unschooled (Table 1).

**Table 1:** Sociodemographic data of study participants

	**Mong** ***n* = 85**	**Nkongmessa** ***n* = 69**	**Nkolkosse** ***n* = 55**	**Overall** ***N* = 209**
Gender: *n* (%)				
Male	42 (49.4)	38 (55.1)	22 (40.0)	102 (48.8)
Female	43 (50.6)	31 (44.9)	33 (60.0)	107 (51.2)
Age in years: median (IQR)	10 (9–12)	10 (7–11)	10 (7–11)	10 (8–12)
Age-groups: *n* (%)				
6–7 years	15 (17.6)	21 (30.4)	16 (29.1)	52 (24.9)
8–9 years	13 (15.3)	13 (18.8)	10 (18.2)	36 (17.2)
10–11 years	27 (31.8)	19 (27.5)	17 (30.9)	63 (30.1)
12–13 years	21 (24.7)	12 (17.4)	11 (20.0)	44 (21.1)
14–16 years	9 (10.6)	4 (5.8)	1 (1.8)	14 (6.7)
Duration* in village: median (IQR)	7 (6–10)	8 (7–10)	6 (4–8.5)	7 (5–10)
Education level: *n* (%)				
None	1 (1.2)	1 (1.5)	0	2 (1.0)
Primary	75 (88.2)	59 (85.5)	55 (100)	189 (90.4)
Secondary	9 (10.6)	9 (13.0)	0	18 (8.6)
Previous ivermectin use: *n* (%)*	66/85 (77.6)	68/69 (98.6)	22/44 (50.0)	156/198 (78.8)

*IQR* interquartile range

*11 missing data

### Results of the Ov16 Testing and Anthropometric Evaluation

**Fig. 2:**
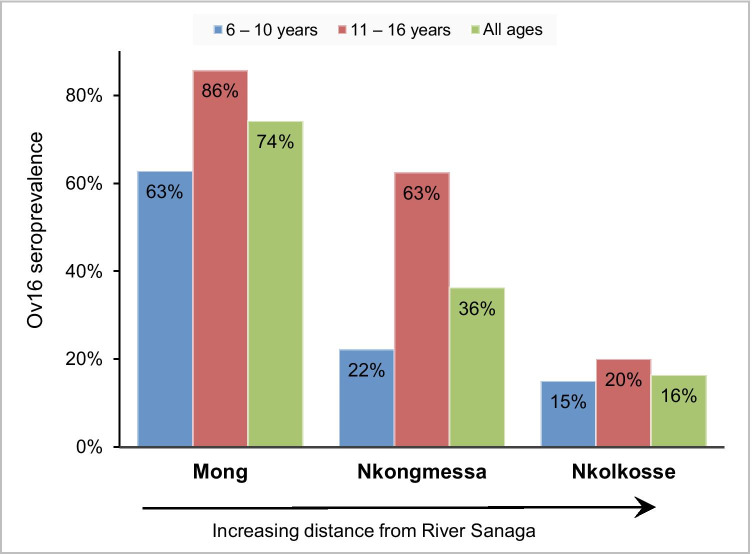
Ov16 seroprevalence in the study villages

Among the participants, 97 (46.4%) were Ov16-positive while 112 (53.6%) were Ov16-negative. In children aged 10 years and below, Ov16 seroprevalence was 33.6% while it reached 66.7% among older children (*p* < 0.001). Ov16-positive participants were older than Ov16-negative children (median ages 11 [IQR 10–12] vs 9 [IQR 7–10]; Mann–Whitney *U p*-value < 0.001). The Ov16 seroprevalence varied significantly across the study villages (*p* < 0.001). Additionally, we observed that the proportion of children with Ov16 antibodies decreased with increasing distance of the village from the Sanaga River (Fig. 2).

The prevalence of stunting was 8.2% among Ov16-positive, and 5.4% among Ov16-negative participants (*p* = 0.578); 10.3% of Ov16-positive participants were underweight, compared to 6.3% children who were underweight in the Ov16-negative group (*p* = 0.284). There was no significant association between Ov16 status and having received ivermectin in the past (chi-squared *p*-value = 0.616).

### Neurocognitive Performance of Participants

The mean neurocognitive scores observed in our study population were lower than those recorded by Ruffieux et al. when validating these tests in Cameroonian school-aged children (Ruffieux et al., 2009) (Supplementary Appendix 2). When comparing the mean neurocognitive scores in Ov16-positive participants (cases) vs Ov16-negative participants (controls), no significant differences were noted (Table 2).

**Table 2:** Comparison of mean neurocognitive scores in Ov16-positive and Ov16-negative participants

	**6–7 years**			**8–9 years**			**10–11 years**			**12–13 years**			**14–16 years**		
	**Ov16 Neg:** Mean (SD) *n* = 37	**Ov16 Pos:** Mean (SD) *n* = 15	***P*-Value:** Mann Whitney *U*	**Ov16 Neg:** Mean (SD) *n* = 28	**Ov16 Pos:** Mean (SD) *n* = 8	***P*-Value:** Mann Whitney U	**Ov16 Neg:** Mean (SD) *n* = 26	**Ov16 Pos:** Mean (SD) *n* = 37	***P*-Value:** Mann Whitney U	**Ov16 Neg:** Mean (SD) *n* = 17	**Ov16 Pos:** Mean (SD) *n* = 27	***P*-Value:** Mann Whitney U	**Ov16 Neg:** Mean (SD) *n* = 4	**Ov16 Pos:** Mean (SD) *n* = 10	***P*-Value:** Mann Whitney *U*
Pegboard, DH	9.38 (2.14)	10.2 (2.27)	0.297	10.9 (1.99)	10.0 (2.98)	0.451	12.0 (1.77)	12.8 (1.69)	0.269	12.7 (1.86)	13.2 (1.59)	0.432	12.2 (1.26)	11.8 (1.14)	0.551
Pegboard, NDH	8.35 (1.70)	8.93 (1.71)	0.180	9.82 (1.74)	9.50 (1.77)	0.714	11.2 (1.30)	11.8 (1.58)	0.109	12.0 (1.46)	12.0 (2.00)	0.804	10.8 (1.26)	11.7 (1.25)	0.271
Pegboard, BH	6.27 (1.66)	6.67 (1.35)	0.395	7.50 (1.69)	7.25 (2.12)	1.000	8.77 (1.42)	8.65 (1.48)	0.647	9.35 (1.17)	9.33 (1.69)	0.881	9.00 (1.63)	9.20 (1.99)	0.773
Digit span, F	6.03 (1.44)	6.00 (1.36)	0.983	6.57 (1.69)	7.00 (2.45)	0.831	6.92 (1.60)	6.97 (1.79)	0.910	6.88 (1.32)	7.44 (1.65)	0.079	9.00 (1.15)	8.50 (3.54)	0.316
Digit span, B	1.24 (1.95)	1.07 (1.91)	0.961	2.93 (2.36)	3.12 (2.10)	0.952	4.35 (1.57)	3.95 (2.00)	0.742	4.59 (1.50)	4.93 (1.59)	0.356	5.25 (1.26)	4.80 (0.79)	0.546
Hand movement	7.89 (2.64)	9.13 (2.53)	0.129	9.89 (3.31)	10.1 (2.59)	0.924	10.7 (2.36)	10.1 (3.06)	0.166	10.8 (1.89)	10.5 (2.89)	0.696	12.0 (1.63)	11.2 (3.49)	0.666
Semantic VF	5.70 (3.85)	5.47 (2.72)	0.831	8.21 (3.35)	5.75 (2.55)	0.082	10.7 (4.58)	9.68 (3.29)	0.542	11.7 (3.77)	10.8 (4.11)	0.338	11.8 (2.22)	10.7 (3.13)	0.520
MMSE	23.2 (3.99)	24.3 (2.64)	0.304	27.2 (3.84)	27.0 (4.07)	0.939	30.2 (3.09)	29.0 (3.83)	0.211	30.7 (2.42)	30.6 (2.28)	0.669	32.8 (0.96)	31.2 (2.70)	0.379
IHDS	6.96 (2.61)	6.47 (2.62)	0.341	7.79 (2.12)	7.38 (1.83)	0.431	8.62 (2.19)	7.76 (2.02)	0.082	9.09 (1.62)	9.19 (1.61)	0.884	10.0 (1.41)	9.10 (1.47)	0.389

*DH* dominant hand, *NDH* non-dominant hand, *BH* Both hands, *F* forward, *B* backward, *VF* verbal fluency, *MMSE* mini-mental state exam, *IHDS* International HIV Dementia Scale, *SD* standard deviation.

Applying the appropriate cutoffs (5th percentile) per age group, no significant differences in the neurocognitive outcomes between cases and controls were observed (Table 3). Sensitivity analysis including only ivermectin-naïve children (*n* = 42) found a similar distribution of children with normal vs abnormal neurocognitive performance among cases and controls (see Supplementary Appendix 3).

**Table 3:** Binary neurocognitive outcomes of participants by Ov16 status

**Neurocognitive tests and outcomes**		**Ov16-negative (controls)** ***n* = 112**	**Ov16-positive (cases)** ***n* = 97**	*P*-Value*
Pegboard, dominant hand	Normal: *n* (%)	108 (96.4%)	94 (96.9%)	1.000
	Below normal: *n* (%)	4 (3.6%)	3 (3.1%)	
Pegboard, non-dominant hand	Normal: *n* (%)	108 (96.4%)	94 (96.9%)	1.000
	Below normal: *n* (%)	4 (3.6%)	3 (3.1%)	
Pegboard, both hands	Normal: *n* (%)	109 (97.3%)	93 (95.9%)	0.707
	Below normal: *n* (%)	3 (2.7%)	4 (4.1%)	
Digit span forward	Normal: *n* (%)	112 (100%)	94 (96.9%)	0.098
	Below normal: *n* (%)	0 (0%)	3 (3.1%)	
Digit span backward	Normal: *n* (%)	111 (99.1%)	95 (97.9%)	0.598
	Below normal: *n* (%)	1 (0.9%)	2 (2.1%)	
Hand movements	Normal: *n* (%)	107 (95.5%)	93 (95.9%)	1.000
	Below normal: *n* (%)	5 (4.5%)	4 (4.1%)	
Semantic verbal fluency	Normal: *n* (%)	109 (97.3%)	89 (91.8%)	0.137
	Below normal: *n* (%)	3 (2.7%)	8 (8.2%)	
Mini-mental state exam	Normal: *n* (%)	108 (96.4%)	92 (94.8%)	0.736
	Below normal: *n* (%)	4 (3.6%)	5 (5.2%)	
International HIV Dementia Scale	Normal: *n* (%)	105 (93.8%)	91 (93.8%)	1.000
	Below normal: *n* (%)	7 (6.2%)	6 (6.2%)	

* Yates-corrected Chi-squared test

### Multivariable Analysis

We ran six multiple linear regression models to investigate the association between standardized neurocognitive outcomes (*z*-scores) and Ov16 status, adjusting for gender, education level, previous ivermectin use, BMI-for-age *z*-score, and height-for-age *z*-score. Of the six neurocognitive tests, two (semantic verbal fluency and IHDS) were significantly associated with Ov16 results: a positive Ov16 serology significantly decreased the neurocognitive scores (Table 4). Of note, an increasing number of previous ivermectin doses was frequently associated with higher neurocognitive scores.

**Table 4:** Multiple linear regression models investigating associations between the neurocognitive tests and Ov16 status

Model No	Covariates	Adjusted regression coefficient (95% CI)	*P*-Value	Model No	Covariates	Adjusted regression coefficient (95% CI)	*P*-Value	Model No	Covariates	Adjusted regression coefficient (95% CI)	P-value
1 (DV average Pegboard score)	Ov16-positive test	0.06 (− 0.21 to 0.33)	0.660	2 (DV average Digit span score)	Ov16-positive test	− 0.02 (− 0.29 to 0.26)	0.907	3 (DV hand movements)	Ov16-positive test	− 0.05 (− 0.33 to 0.22)	0.705
	Female gender	0.02 (− 0.24 to 0.29)	0.860		Female gender	− 0.11 (− 0.38 to 0.16)	0.428		Female gender	− 0.35 (− 0.62 to − 0.78)	0.012
	Previous IVM 0	0.44 (–21 to 1.08)	0.182		Previous IVM 0	0.92 (0.26 to 1.57)	0.006		Previous IVM 0	0.71 (0.06 to 1.36)	0.032
	Previous IVM 1–2 ×	0.06 (–0.54 to 0.66)	0.840		Previous IVM 1–2 ×	0.63 (0.02 to 1.24)	0.044		Previous IVM 1–2 ×	0.38 (− 0.23 to 0.99)	0.219
	Previous IVM 3–5 ×	0.40 (–0.24 to 1.04)	0.215		Previous IVM 3–5 ×	0.58 (− 0.07 to 1.23)	0082		Previous IVM 3–5 ×	0.50 (− 0.14 to 1.15)	0.126
	Previous IVM > 5 ×	0.30 (–0.53 to 1.13)	0.471		Previous IVM > 5 ×	0.58 (− 0.26 to 1.43)	0.176		Previous IVM > 5 ×	0.80 (− 0.04 to 1.64)	0.061
	Education: primary	2.02 (0.67 to 3.37)	0.004		Education: primary	0.64 (− 0.74 to 2.01)	0.363		Education: primary	1.19 (− 0.18 to 2.55)	0.088
	Education: secondary	2.09 (0.66 to 3.52)	0.004		Education: secondary	1.18 (− 0.28 to 2.64)	0.113		Education: secondary	1.28 (− 0.17 to 2.73)	0.082
	BMI-for-age *z*-score	–0.08 (–0.19 to 0.03)	0.142		BMI-for-age *z*-score	0.08 (− 0.03 to 0.20)	0.154		BMI-for-age *z*-score	0.08 (− 0.04 to 0.19)	0.176
	Height-for-age *z*-score	0.13 (0.02 to 0.24)	0.020		Height-for-age *z*-score	0.06 (− 0.05 to 0.18)	0.260		Height-for-age *z*-score	0.10 (− 0.01 to 0.21)	0.079
4 (DV semantic verbal fluency)	Ov16-positive test	− 0.38 (− 0.65 to − 0.11)	0.006	5 (DV MMSE)	Ov16-positive test	− 0.21 (− 0.48 to 0.06)	0.123	6 (DV IHDS)	Ov16-positive test	− 0.31 (− 0.56 to − 0.04)	0.025
	Female gender	− 0.10 (− 0.37 to 0.16)	0.442		Female gender	− 0.14 (− 0.41 to 0.12)	0.287		Female gender	− 0.04 (− 0.30 to 0.23)	0.777
	Previous IVM 0	0.72 (0.08 to 1.36)	0.028		Previous IVM 0	0.76 (0.11 to 1.40)	0.021		Previous IVM 0	0.45 (− 0.19 to 1.09)	0.165
	Previous IVM 1–2 ×	0.47 (− 0.13 to 1.08)	0.121		Previous IVM 1–2 ×	0.64 (0.04 to 1.24)	0.036		Previous IVM 1–2 ×	0.60 (0.01 to 1.20)	0.048
	Previous IVM 3–5 ×	0.65 (0.01 to 1.29)	0.046		Previous IVM 3–5 ×	0.70 (0.06 to 1.34)	0.032		Previous IVM 3–5 ×	0.76 (0.13 to 1.40)	0.019
	Previous IVM > 5 ×	1.27 (0.44 to 2.10)	0.003		Previous IVM > 5 ×	1.05(0.22 to 1.88)	0.013		Previous IVM > 5 ×	1.39 (0.56 to 2.21)	0.001
	Education: primary	1.61 (0.26 to 2.95)	0.019		Education: primary	1.46 (0.11 to 2.81)	0.033		Education: primary	0.95 (− 0.39 to 2.29)	0.114
	Education: secondary	2.01 (0.59 to 3.44)	0.006		Education: secondary	1.90 (0.47 to 3.33)	0.009		Education: secondary	1.14 (− 0.28 to 2.57)	
	BMI-for-age *z*-score	− 0.02 (− 0.13 to 0.09)	0.727		BMI-for-age *z*-score	− 0.00 (− 0.11 to 0.11)	0.999		BMI-for-age *z*-score	0.05 (− 0.06 to 0.16)	0.406
	Height-for-age *z*-score	0.01 (− 0.10 to 0.12)	0.802		Height-for-age *z*-score	0.16 (0.05 to 0.27)	0.004		Height-for-age *z*-score	0.16 (0.05 to 0.27)	0.004

*DV* dependent variable, *IVM* ivermectin

## Discussion

As far as we know, this is the first study providing evidence of a possible association between neurocognitive performance and infection with *O. volvulus.* This association may not be apparent using dichotomized neurocognitive test scores but becomes more apparent when neurocognitive performance is appreciated on a continuous scale. Moreover, a proper interpretation of these scores on a continuous scale necessitates prior adjustments for the participants’ age before introducing them in a multivariable model because both neurocognitive performance and Ov16 status are strongly age-dependent (Gardner & Broman, 1979; Golden et al., 2016). The multivariable analyses showed that a positive Ov16 serology is associated with lower age-adjusted scores on the semantic verbal fluency and IHDS tests, suggesting that exposure to *O. volvulus* may impact neurocognitive performance at least to a certain extent. This certainly requires more in-depth investigations including a prospective study of exposed children to evaluate their neurocognitive evolution later in life.

Although the gold standard diagnostic approach for onchocerciasis is the detection of *O. volvulus* microfilariae in skin snips, in this study, we used the Ov16 RDT to diagnose past or ongoing onchocercal infection in our participants. While Ov16 tests cannot inform about the timing of *O. volvulus* infection, they are very reliable for detecting individuals who have been inoculated with the parasite at some point in their life (Weil GJ et al. 2000). Bearing in mind that even a past infection with *O. volvulus* during early childhood was found to increase the risk of developing epilepsy later in life (Chesnais et al., 2018, 2020), it is equally plausible that a past exposure to the parasite, as detected by the Ov16 test, could influence neurocognitive performance several years later. It is not excluded that a reduced cognitive performance may even be a precursor to the development of onchocerciasis-related seizures and epilepsy; this may explain why persons with NS (considered as a severe form of OAE with high *O. volvulus* infection intensity) often experience profound mental impairment (Abd-Elfarag et al., 2020).

Our study confirms the feasibility of administering a battery of neurocognitive tests in a remote, resource-limited setting in Cameroon. We must however note that the mean neurocognitive scores of our participants, recruited from rural Cameroon, were slightly lower compared to the normative data which was obtained from children residing in the urban city of Yaoundé (Ruffieux et al., 2009) (see Supplementary Appendix 2). Such rural–urban disparities had previously been noted (Hermida et al., 2019) and suggest that children’s cognitive performance can be influenced to an extent by the milieu in which they grow up. Conducting neurocognitive cognitive studies in such rural settings poses several logistical challenges, as well as methodological issues because of additional confounders that may not be present in urban settings where the normative neurocognitive data were collected. The fact that we generated standardized scores based on our own data (and not the normative data provided by Ruffieux et al.) makes our analysis more robust and relevant for our study population.

The multivariable models in which neurocognitive scores were introduced as standardized continuous variables revealed that being Ov16-positive was associated with lower scores on the IHDS scale which was used in this study as a proxy for encephalopathy, given that children do not develop dementia per se. Unlike other tests which measure only a specific neurocognitive function, IHDS has components of psychomotor speed evaluation and memory assessment. A comparison of the performances of IHDS and MMSE in an adult Nigerian population confirmed the superiority of the IHDS in diagnosing HIV-associated neurocognitive disorders (Oshinaike et al., 2012). Besides the IHDS, only the semantic verbal fluency test (which assesses mental flexibility) was also associated with Ov16 serology. Based on these results, it appears that infection with *O. volvulus* may impact executive functions more than motor functions. In line with these observations, a recent study conducted in the onchocerciasis-endemic village of Bilomo in Cameroon found a high prevalence of both executive function deficits (92.5% among PWE vs 40.0% among controls; *p* < 0.001) and decreased verbal fluency (100% among PWE vs 45% among controls; *p* < 0.001), with a longer duration of residence in the village being associated with poorer neurocognitive performance (Njamnshi et al., 2020). This suggests that exposure to *O. volvulus* may induce cognitive impairment which could be exacerbated by the development of epilepsy. More tests assessing executive function are needed to further explore these preliminary findings, bearing in mind that the verbal fluency test is language-level-dependent, meaning that, if a child has a lower vocabulary level, he/she will perform lower at this test (and maybe not because of lower flexibility).

The multivariable analysis also showed that an increasing frequency of past ivermectin use was consistently associated with better cognitive outcomes in models 4, 5, and 6 (Table 4). This suggests that, by frequently reducing the *O. volvulus* microfilarial load using ivermectin, children may be prevented from developing some form of neurocognitive impairment. This concurs with previous observations in South Sudan which demonstrated that PWE with higher *O. volvulus* parasitic load were often more cognitively impaired and had higher disability scores compared to other PWE with milder infection (Abd-Elfarag et al., 2020). It was however difficult to conclude about the timing of cognitive impairment (whether it preceded seizure onset or not) given the cross-sectional nature of that study.

The Ov16 seroprevalence was 46.4% among all participants and 33.6% in children aged 10 years and below; this is indicative of high ongoing onchocerciasis transmission in the study villages. Similar to our findings, a greater onchocerciasis burden in villages closer to the river (blackfly breeding site) was earlier reported in Cameroon by Mendoza Aldana et al. (Mendoza Aldana et al., 1997). Boussinesq et al. (Boussinesq et al. 2002) further demonstrated that, in addition to the onchocerciasis burden, the prevalence of epilepsy also increases with decreasing distance from the river. Therefore, stepping up onchocerciasis elimination efforts in such areas will minimize the risk for onchocerciasis and related neurological disorders in the exposed communities. The fact that about one-fifth of the participants had never received ivermectin underscores the need for interventions to increase the effectiveness of community-directed treatment with ivermectin in the study villages. Alternative treatment strategies such as test-and-not-treat approaches would also benefit these communities where onchocerciasis is co-endemic with loiasis (Boussinesq et al., 2018; Kamgno et al., 2017).

This pilot study provides the first empirical data of neurocognitive performance associated with onchocerciasis exposure status. As major limitations, we did not perform skin snips to confirm active onchocercal infection and quantify the parasitic load, nor electroencephalograms (EEG) to exclude persons with sub-clinical epilepsy. We also did not do laboratory investigations to rule out malaria, anemia, or infection with intestinal worms as these conditions may influence cognitive performance (Kihara et al., 2006; Wieringa et al., 2011). Participants were not tested for loiasis, which is co-endemic in the study sites and can result in encephalopathy during ivermectin treatment (Gardon et al., 1997). Furthermore, our data analysis did not adjust for socio-economic factors which have previously been associated with neurocognitive development in children (Hermida et al., 2019).

In conclusion, our findings suggest that the neurocognitive performance of school-aged children in the Lekié division of Cameroon may be impacted by previous exposure to *O. volvulus*. More comprehensive and longitudinal studies are needed to ascertain these preliminary observations.

## Supplementary Information

Below is the link to the electronic supplementary material.

Supplementary file1 (DOCX 15 KB)

Supplementary file2 (DOCX 14 KB)

Supplementary file3 (DOCX 14 KB)

## References

[CR1] Abd-Elfarag G, Carter JY, Raimon S, Sebit W, Suliman A, Fodjo JNS (2020). Persons with onchocerciasis-associated epilepsy and nodding seizures have a more severe form of epilepsy with more cognitive impairment and higher levels of Onchocerca volvulus infection.. Epileptic Disorders : International Epilepsy Journal with Videotape.

[CR2] Boussinesq M, Fobi G, Kuesel AC (2018). Alternative treatment strategies to accelerate the elimination of onchocerciasis.. International Health.

[CR3] Boussinesq M, Pion SD, Ngangue D, Kamgno J (2002). Relationship between onchocerciasis and epilepsy: A matched case-control study in the Mbam Valley, Republic of Cameroon.. Transactions of the Royal Society of Tropical Medicine and Hygiene.

[CR4] Burnham G (1998). Onchocerciasis.. Lancet.

[CR5] Chesnais, C. B., Bizet, C., Campillo, J. T., Njamnshi, W. Y., Bopda, J., Nwane, P., et al. (2020). A second population-based cohort study in Cameroon confirms the temporal relationship between onchocerciasis and epilepsy. *Open Forum Infectious Diseases*, ofaa206. 10.1093/ofid/ofaa206. 10.1093/ofid/ofaa206 PMC7304933 32587878

[CR6] Chesnais CB, Nana-Djeunga HC, Njamnshi AK, Lenou-Nanga CG, Boullé C, Bissek ACZK (2018). The temporal relationship between onchocerciasis and epilepsy: A population-based cohort study.. The Lancet Infectious Diseases.

[CR7] Colebunders R, Abd-Elfarag G, Carter JY, Olore PC, Puok K, Menon S (2018). Clinical characteristics of onchocerciasis-associated epilepsy in villages in Maridi County, Republic of South Sudan.. Seizure.

[CR8] Colebunders R, SieweFodjo JN, Hotterbeekx A (2018). Onchocerciasis-associated epilepsy, an additional reason for strengthening onchocerciasis elimination programs.. Trends in Parasitology.

[CR9] de Onis M (2007). Development of a WHO growth reference for school-aged children and adolescents.. Bulletin of the World Health Organization.

[CR10] Föger K, Gora-Stahlberg G, Sejvar J, Ovuga E, Jilek-Aall L, Schmutzhard E (2017). Nakalanga syndrome: Clinical characteristics, potential causes, and its relationship with recently described nodding syndrome.. PLOS Neglected Tropical Diseases.

[CR11] Gardner RA, Broman M (1979). The Purdue Pegboard: Normative data on 1334 school children.. Journal of Clinical Child Psychology.

[CR12] Gardon J, Gardon-Wendel N, Demanga-Ngangue, Kamgno J, Chippaux JP, Boussinesq M (1997). Serious reactions after mass treatment of onchocerciasis with ivermectin in an area endemic for Loa loa infection.. The Lancet.

[CR13] Golden A, Faulx D, Kalnoky M, Stevens E, Yokobe L, Peck R (2016). Analysis of age-dependent trends in Ov16 IgG4 seroprevalence to onchocerciasis.. Parasites & Vectors.

[CR14] Hermida MJ, Shalom DE, Segretin MS, Goldin AP, Abril MC, Lipina SJ, Sigman M (2019). Risks for child cognitive development in rural contexts.. Frontiers in Psychology.

[CR15] Idro R, Namusoke H, Abbo C, Mutamba BB, Kakooza-Mwesige A, Opoka RO (2014). Patients with nodding syndrome in Uganda improve with symptomatic treatment: A cross-sectional study.. British Medical Journal Open.

[CR16] Idro R, Ogwang R, Kayongo E, Gumisiriza N, Lanyero A, Kakooza-Mwesige A, Opar B (2018). The natural history of nodding syndrome.. Epileptic Disorders.

[CR17] Kamgno J, Pion SD, Chesnais CB, Bakalar MH, D’Ambrosio MV, Mackenzie CD (2017). A test-and-not-treat strategy for Onchocerciasis in *Loa loa* –endemic areas.. New England Journal of Medicine.

[CR18] Kihara M, Carter JA, Newton CRJC (2006). The effect of *Plasmodium falciparum* on cognition: A systematic review: *Plasmodium falciparum* and cognition.. Tropical Medicine & International Health.

[CR19] Mendoza Aldana J, Piechulek H, Maguire J (1997). Forest onchocerciasis in Cameroon: Its distribution and implications for selection of communities for control programmes.. Annals of Tropical Medicine & Parasitology.

[CR20] Moura R, Andrade PMO, Fontes PLB, Ferreira FO, de Souza Salvador L, Carvalho MRS, Haase VG (2017). Mini-mental state exam for children (MMC) in children with hemiplegic cerebral palsy.. Dementia & Neuropsychologia.

[CR21] Njamnshi AK, Chokote E-S, Ngarka L, Nfor LN, Tabah EN, Atchou JGB (2020). Epilepsy-associated neurocognitive disorders (EAND) in an onchocerciasis-endemic rural community in Cameroon: A population-based case–control study.. Epilepsy & Behavior.

[CR22] Njamnshi AK, de Paul Djientcheu V, Fonsah JY, Yepnjio FN, Njamnshi DM, Muna WF (2008). The International HIV Dementia Scale is a useful screening tool for HIV-associated dementia/cognitive impairment in HIV-infected adults in Yaoundé-Cameroon.. JAIDS Journal of Acquired Immune Deficiency Syndromes.

[CR23] Oshinaike OO, Akinbami AA, Ojo OO, Ojini IF, Okubadejo UN, Danesi AM (2012). Comparison of the Minimental State Examination Scale and the International HIV Dementia Scale in Assessing Cognitive Function in Nigerian HIV Patients on Antiretroviral Therapy.. AIDS Research and Treatment.

[CR24] Pion SD, Kaiser C, Boutros-Toni F, Cournil A, Taylor MM, Meredith SEO, et al. (2009). Epilepsy in Onchocerciasis endemic areas: Systematic review and meta-analysis of population-based surveys. *PLoS Neglected Tropical Diseases*, *3*(6). 10.1371/journal.pntd.0000461. 10.1371/journal.pntd.0000461 PMC2691484 19529767

[CR25] Raper AB, Ladkin RG (1950). Endemic dwarfism in Uganda.. East African Medical Journal.

[CR26] Ruffieux N, Njamnshi AK, Mayer E, Sztajzel R, Eta SC, Doh RF (2009). Neuropsychology in Cameroon: First normative data for cognitive tests among school-aged children.. Child Neuropsychology.

[CR27] Ruffieux N, Njamnshi AK, Wonkam A, Hauert C-A, Chanal J, Verdon V (2013). Association between biological markers of sickle cell disease and cognitive functioning amongst Cameroonian children.. Child Neuropsychology.

[CR28] Sacktor NC, Wong M, Nakasujja N, Skolasky RL, Selnes OA, Musisi S (2005). The International HIV Dementia Scale: A new rapid screening test for HIV dementia.. AIDS (london, England).

[CR29] Siewe Fodjo, J. N., Dekker, M. C. J., Idro, R., Mandro, M. N., Preux, P.-M., Njamnshi, A. K., & Colebunders, R. (2019). Comprehensive management of epilepsy in onchocerciasis-endemic areas: Lessons learnt from community-based surveys. *Infectious Diseases of Poverty*, *8*(1). 10.1186/s40249-019-0523-y. 10.1186/s40249-019-0523-y PMC6368958 30738437

[CR30] Siewe Fodjo JN, Mandro M, Mukendi D, Tepage F, Menon S, Nakato S (2019). Onchocerciasis-associated epilepsy in the Democratic Republic of Congo: Clinical description and relationship with microfilarial density.. PLOS Neglected Tropical Diseases.

[CR31] Siewe Fodjo JN, Ngarka L, Tatah G, Mengnjo MK, Nfor LN, Chokote ES (2019). Clinical presentations of onchocerciasis-associated epilepsy (OAE) in Cameroon.. Epilepsy & Behavior.

[CR32] Weil GJ, Steel C, Liftis F, Li B, Mearns G, Lobos E, Nutman TB (2000). A rapid-format antibody card test for diagnosis of onchocerciasis.. Journal of Infectious Diseases.

[CR33] Wieringa FT, Nga TT, Winichagoon P, Wasantwisut E, Dijkhuizen MA, Khan NC (2011). Decreased parasite load and improved cognitive outcomes caused by deworming and consumption of multi-micronutrient fortified biscuits in rural Vietnamese schoolchildren.. The American Journal of Tropical Medicine and Hygiene.

