# Supplementary material for: Association Between Ov16 Seropositivity and Neurocognitive Performance Among Children in Rural Cameroon: a Pilot Study
Source: J Pediatr Neuropsychol. 2021 Sep 7;7(4):192–202. doi: 10.1007/s40817-021-00111-z (PMC8602181; doi:10.1007/s40817-021-00111-z)
Supplement: Supplementary file 1 — Supplementary file1 (DOCX 15 KB) [file 40817_2021_111_MOESM1_ESM.docx]

**Appendix 1: Summary procedures for neurocognitive assessment**

| **Cognitive test** | **Procedures** | **Remarks** |
| --- | --- | --- |
| Purdue Pegboard test | This is a manual dexterity test that involves sequential insertion of pegs into small holes on a pre-designed board. We administered this test under three conditions: dominant hand, non-dominant hand, and both hands (30 seconds each). The number of pegs inserted was noted each time. | We omitted the “assembly” step for practical reasons. A score was considered as failed if below the cut-off defined as the 5^th^ percentile score for each age group. |
| Digit span | This test primarily assesses auditory short-term memory and working memory. A sequence of numbers is read to the child, who is asked to repeat the numbers in same order (Digit Span Forward). The child is then asked to repeat the sequence of number in reverse order (Digit Span Backward). Each successful attempt earns the child 1 point. | No modifications. A score was considered as failed if below the cut-off defined as the 5^th^ percentile score for each age group. |
| Hand movements | This test measures predominantly visual attention and working memory. The examiner taps out a sequence on the table, using either fist, palm, or side of hand. The child must then imitate the same sequence. The number of correct sequences imitated is recorded. | No modifications. A score was considered as failed if below the cut-off defined as the 5^th^ percentile score for each age group. |
| Semantic verbal fluency | The child is asked to name as many animals as possible (including fishes, birds) in one minute. The number of correct answers is recorded. | No modifications. A score was considered as failed if below the cut-off defined as the 5^th^ percentile score for each age group. |
| Mini-mental test | Administration of a short mini-mental state exam for children questionnaire [published by Moura et al. (Moura et al., 2017)] | We excluded the questions requiring “reading” and “writing,” yielding a new total of 35. A score was considered as failed if below the cut-off defined as the 5^th^ percentile score for each age group. |
| International HIV Dementia Scale (IHDS) | Three sub-tests were administered, each scored on a scale ranging from 0–4: timed finger-tapping (motor speed); timed alternating hand sequence (psychomotor speed); and recall of 4 items at 2 minutes interval (memory registration and recall). | The IHDS adaptations published by Njamnshi et al. (Njamnshi et al., 2008) were applied. A score was considered as failed if below the cut-off defined as the 5^th^ percentile score for each age group. |

**References**

Moura R, Andrade PMO, Fontes PLB, Ferreira FO, Salvador L de S, Carvalho MRS, et al. Mini-mental state exam for children (MMC) in children with hemiplegic cerebral palsy. Dementia & Neuropsychologia 2017;11:287–96. https://doi.org/10.1590/1980-57642016dn11-030011.

Njamnshi AK, Djientcheu V de P, Fonsah JY, Yepnjio FN, Njamnshi DM, Muna WF. The International HIV Dementia Scale Is a Useful Screening Tool for HIV-Associated Dementia/Cognitive Impairment in HIV-Infected Adults in Yaoundé-Cameroon: JAIDS Journal of Acquired Immune Deficiency Syndromes 2008;49:393–7. https://doi.org/10.1097/QAI.0b013e318183a9df.
