# Supplementary material for: Association Between Ov16 Seropositivity and Neurocognitive Performance Among Children in Rural Cameroon: a Pilot Study
Source: J Pediatr Neuropsychol. 2021 Sep 7;7(4):192–202. doi: 10.1007/s40817-021-00111-z (PMC8602181; doi:10.1007/s40817-021-00111-z)
Supplement: Supplementary file 2 — Supplementary file2 (DOCX 14 KB) [file 40817_2021_111_MOESM2_ESM.docx]

**Appendix 2: Normative mean scores and study mean scores for the neurocognitive tests**

| **Age groups:** | **6–7 years** | | **8–9 years** | | **10–11 years** | | **12–13 years** | | **14–16 years** | |
| --- | --- | --- | --- | --- | --- | --- | --- | --- | --- | --- |
|  | Normative* Mean (SD)  *n = 20* | This study: Mean (SD)  *n = 52* | Normative* Mean (SD)  *n = 20* | This study: Mean (SD)  *n = 36* | Normative* Mean (SD)  *n = 20* | This study: Mean (SD)  *n = 63* | Normative* Mean (SD)  *n = 22* | This study: Mean (SD)  *n = 44* | Normative* Mean (SD)  *n = 22* | This study: Mean (SD)  *n = 14* |
| Pegboard, dominant hand | 10.9 (2.0) | 9.6 (2.19) | 12.2 (1.0) | 10.7 (2.23) | 12.5 (1.4) | 12.4 (1.75) | 13.7 (1.3) | 13.0 (1.70) | 13.5 (1.9) | 11.9 (1.14) |
| Pegboard, non-dominant hand | 9.1 (2.0) | 8.5 (1.71) | 10.6 (1.3) | 9.8 (1.73) | 11.4 (1.2) | 11.6 (1.49) | 13.0 (1.5) | 12.0 (1.79) | 12.8 (2.2) | 11.4 (1.28) |
| Pegboard, both hands | 7.6 (1.5) | 6.4 (1.57) | 8.5 (1.5) | 7.4 (1.76) | 9.4 (1.4) | 8.7 (1.44) | 10.2 (1.7) | 9.3 (1.49) | 9.8 (1.4) | 9.1 (1.83) |
| Digit span, Forward | 6.2 (1.7) | 6.0 (1.41) | 6.1 (1.7) | 6.7 (1.85) | 6.5 (1.4) | 7.0 (1.70) | 7.5 (1.8) | 7.2 (1.54) | 8.3 (1.9) | 8.6 (3.00) |
| Digit span, Backward | 4.8 (0.9) | 1.2 (1.92) | 4.9 (1.3) | 3.0 (2.27) | 5.6 (1.4) | 4.1 (1.83) | 6.7 (1.7) | 4.8 (1.55) | 6.5 (1.7) | 4.9 (0.92) |
| Hand movements | 8.7 (2.5) | 8.3 (2.65) | 9.1 (3.2) | 9.9 (3.13) | 11.1 (2.7) | 10.3 (2.79) | 12.9 (3.3) | 10.6 (2.53) | 13.1 (3.0) | 11.4 (3.03) |
| Semantic Verbal Fluency | 8.5 (3.7) | 5.6 (3.54) | 10.8 (5.7) | 7.7 (3.32) | 12.3 (3.6) | 10.1 (3.88) | 14.8 (5.4) | 11.2 (3.96) | 16.1 (3.6) | 11.0 (2.86) |
| *SD: Standard deviation*  **Normative data of Cameroonian children reported by Ruffieux et al:* ***Neuropsychology in Cameroon: First Normative Data for Cognitive Tests among School-Aged Children****. Child Neuropsychology. 2009 Dec 21;16(1):1–19* | | | | | | | | | | |
