# Supplementary material for: Association Between Ov16 Seropositivity and Neurocognitive Performance Among Children in Rural Cameroon: a Pilot Study
Source: J Pediatr Neuropsychol. 2021 Sep 7;7(4):192–202. doi: 10.1007/s40817-021-00111-z (PMC8602181; doi:10.1007/s40817-021-00111-z)
Supplement: Supplementary file 3 — Supplementary file3 (DOCX 14 KB) [file 40817_2021_111_MOESM3_ESM.docx]

**Supplementary Appendix 3**

**Dichotomized neurocognitive outcomes in ivermectin-naïve children**

| **Neurocognitive tests and outcomes** | | **Ov16-negative (controls)**  **n = 24** | **Ov16-positive (cases)**  **n = 18** | **P-value*** |
| --- | --- | --- | --- | --- |
| Pegboard, dominant hand | Normal: n (%) | 23 (95.8%) | 18 (100%) | 1.000 |
|  | Below normal: n (%) | 1 (4.2%) | 0 (0%) |  |
| Pegboard, non-dominant hand | Normal: n (%) | 23 (95.8%) | 18 (100%) | 1.000 |
|  | Below normal: n (%) | 1 (4.2%) | 0 (0%) |  |
| Pegboard, both hands | Normal: n (%) | 23 (95.8%) | 18 (100%) | 1.000 |
|  | Below normal: n (%) | 1 (4.2%) | 0 (0%) |  |
| Digit span forward | Normal: n (%) | 24 (100%) | 18 (100%) | 1.000 |
|  | Below normal: n (%) | 0 (0%) | 0 (0%) |  |
| Digit span backward | Normal: n (%) | 24 (100%) | 18 (100%) | 1.000 |
|  | Below normal: n (%) | 0 (0%) | 0 (0%) |  |
| Hand movements | Normal: n (%) | 24 (100%) | 17 (94.4%) | 0.429 |
|  | Below normal: n (%) | 0 (0%) | 1 (5.6%) |  |
| Semantic verbal fluency | Normal: n (%) | 23 (95.8%) | 17 (94.4%) | 1.000 |
|  | Below normal: n (%) | 1 (4.2%) | 1 (5.6%) |  |
| Mini-mental state exam | Normal: n (%) | 23 (95.8%) | 18 (100%) | 1.000 |
|  | Below normal: n (%) | 1 (4.2%) | 0 (0%) |  |
| International HIV Dementia Scale | Normal: n (%) | 22 (91.7%) | 18 (100%) | 0.498 |
|  | Below normal: n (%) | 2 (8.3%) | 0 (0%) |  |
| **Yates corrected Chi-squared test* | | | | |
